# COVID-19 and Telenutrition: Remote Consultation in Clinical Nutrition Practice

**DOI:** 10.1093/cdn/nzaa124

**Published:** 2020-12-26

**Authors:** Doaa Farid

**Affiliations:** Department of Family Medicine, McGill University, Montreal, Canada; Centre for Outcomes Research and Evaluation, Research Institute of the McGill University Health Centre, Montreal, Canada

**Keywords:** telehealth, coronavirus disease 2019, health care, dietetics, nutrition, telemedicine

## Abstract

During the coronavirus disease 2019 pandemic, clinical dietitians, as other clinicians, have had to shift their elective in-person clinical encounters to online consultations. Adequate planning and use of tools are essential to minimize delay in delivering medical nutrition therapy to existing and new clients. This article describes the steps required to launch a successful e-nutrition clinic during these times of crisis.

During Covid-19 pandemic, clinical dietitians have had to shift their elective in-person clinical encounters to online consultations. This article describes the steps required to launch a successful telenutrition clinic.

The coronavirus disease 2019 (COVID-19) has caused fear in the globalized world and is a threat that has yet to be well studied (1). In the United States, it has been reported that virtual consultation has increased 10-fold (2). During the COVID-19 pandemic, clinical dietitians have had to shift from in-person client interactions to telenutrition consultations, which comes with its sets of struggles. Virtual consultations through telenutrition are remote technology-supported (video or audio) visits to deliver nutritional therapy (nutritional assessment, analysis, management plan, and follow-up) to clients (3). This article presents four essential steps to successfully shift dietitians’ clinical practice into an integrated “e-nutrition clinic” (**Figure 1**). For those who practice as employees of a health care system or facility, it is important to establish written authorization and appropriate administrative clearances prior to launching independent telenutrition services. This article does not discuss clinical eventualities because the management of clinical patients is subject to varying official guidelines in different regions worldwide.

**FIGURE 1:**
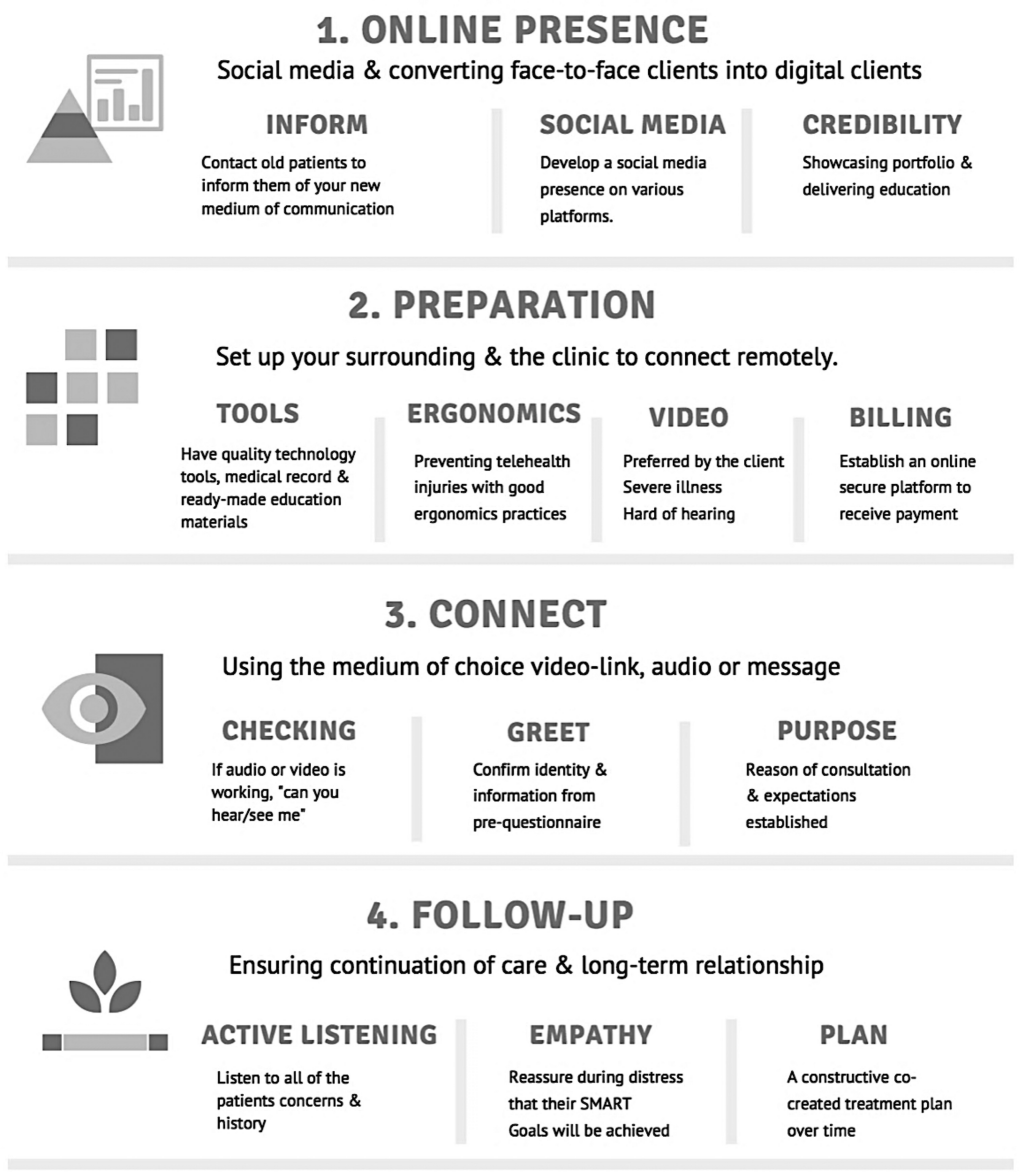
Process of establishing an e-nutrition clinic. SMART, specific, measurable, attainable, relevant, time-limited.

## Step 1: Online Presence

Maintaining an online presence post–COVID-19 is necessary to connect with existing and new clients as well as to promote accurate health information and healthy behaviors (4). Social media is a medium in which relationships are built and continued (**Table 1**). This presents an opportunity to attract clients who are new to telenutrition consultations of any type and may be more likely to attempt to use telemedicine for initial consultations than in the past. Depending on the age and interests of potential or existing clients, some clinicians have established their clinical relationship through a presence on public-facing social media such as TikTok (for younger generations), Instagram, Facebook, and telemedicine platforms and websites (5). These are used to connect to video conferencing communication tools (Skype, Facebook Messenger, WhatsApp, and telemedicine tools such as TabibOnline and Doxy.me) (6). However, these tools need to follow privacy and security measures to be safe for clinical encounters. For example, in the United States, for the consultation to be covered by Medicare or Medicaid benefits, other restrictions are imposed on these types of technologies (7). Video conferencing must be compliant with the Health Insurance Portability and Accountability Act of 1996 (HIPAA). Table 1 presents the platforms that are HIPAA compliant (8).

**TABLE 1:** Video conferencing tools and telemedicine platforms used for virtual consultations^1^

Video conferencing tools for virtual consultations with clients
Apple FaceTime
Facebook Messenger video chat
Google Hangouts video
WhatsApp video chat
Zoom
Skype
HIPAA-compliant platforms made for telemedicine management^2^
Skype for Business/Microsoft Teams
Updox
VSee
Zoom for Healthcare
Doxy.me
Google G Suite Hangouts Meet
Cisco Webex Meetings/Webex Teams
Amazon Chime
GoToMeeting
Spruce Health Care Messenger
Tabibonline.com

1 COVID-19, coronavirus disease 2019; HIPAA, Health Insurance Portability and Accountability Act of 1996.

2 Adapted from Office for Civil Rights’ resources on telehealth and HIPAA during the COVID-19 nationwide public health emergency (7).

## Step 2: Preparation

Preparation for the virtual consultation involves the following: *1*) technical needs and ergonomics, *2*) medical records, *3*) confidentiality, *4*) establishing the environment, *5*) preconsultation preparation, and *6*) pricing and billing.

### Technical needs and ergonomics

Technology tools and ergonomics are important to take into consideration before the encounter. Indeed, the quality of the camera resolution, screen width, and internet connection can impact the interaction. The clinician should verify that the audio and video are working properly before greeting clients. Also, telemedicine injuries can occur; hence, good ergonomics (stand-up desks and good posture) can help prevent shoulder pain, headaches, strains, and hand injuries (9).

### Medical records

Electronic medical records and remote access to existing records will aid in writing clinical notes in a secure platform as well as ensure continuity of care. Dictation systems aid the writing process during telemedicine (10). Some telemedicine platforms also provide an embedded medical records option (Table 1).

### Confidentiality

Confidentiality standards are the same as those in a clinical setting. It is important to mention during the visit that the encounter between the client and the provider is confidential and safe. A mask in view during the consultation (although not worn while speaking) suggests a more clinical or hospital setting, much as a lab coat might (8).

### Establishing the environment

There are three methods of consultation in e-nutrition clinics: messaging, audio, and video calls. Phone conversations can be sufficient for nutritional consultation; however, depending on the client's preference and budget, video consultation might enhance the client–clinician relationship through eye contact and therapeutic presence—that is, it provides additional visual information and diagnostic clues (11–13). Current video conferencing tools likely to be in use by potential clientele include Zoom, Google Duo, Google Meet, FaceTime, and Facebook Messenger Rooms (Table 1) (7). Established telemedicine platforms may also be used (Table 1). Practitioners may wish to discuss their available tools or telemedicine platforms with potential clients in advance so that they can prepare functional and secure connections.

### Preconsultation preparation

Standard preconsultation documents, such as education materials, guidelines for online consultation, frequently asked questions, a referral list to other specialists, and an online registration form, are important to optimize the online encounter. They should be presented in a well-designed format that can be shown during the consultation for visual explanation or sent to the clients pre- or post-consultation. These online tools help clients follow medical nutrition therapy from afar. The guidelines for online consultation includes the process of online consultation, security issues, privacy and confidentiality, and the etiquette of an e-nutrition clinic. In the secure registration form, clinicians can collect information regarding the client's background description, insurance information, brief history, anthropometrics, available laboratory work, and reason for consultation. It is useful for the clinicians to attain a certain level of treatment expectations and client's history to identify the reason for consultation and to analyse recent labs or body composition measurements prior to the online encounter.

### Pricing and billing

Depending on the method of consultation, time spent, and services offered, billing options are established for insured and non-insured clients. Billing will vary according to country and medical payment/insurance systems, but it would be wise to prepare a clear summary of costs and payment options for new clients at the outset for an initial consultation before they incur charges. The method of payment collection should be easy, secure, and bill-generating.

## Step 3: Connect

When talking to clients, it is important to be in a quiet and private area. After greeting the client, their identity and that of the clinician can be verified over the video call or through the document attached in the pre-questionnaire. Then, the client and the clinician can proceed through the standard clinical consultation. Clinicians can use available tests in clients’ homes to further engage the clients. Depending on the patients’ disease management, tools such as home scales, waist circumference measuring tape, and blood glucose tests can be helpful, for example, to assess the progress of weight and diabetes management. During the virtual meeting, clients need to be reassured that they are receiving the same care as they would receive during a face-to-face interaction. The reason for consultation and expectations should be established at the start of the consultation.

## Step 4: Follow-Up

It is important to build rapport with the patient during and after the e-nutrition clinic visit. Active listening and empathy are key to building a healthy interaction with a long-term follow-up (14). A co-created treatment plan with the client using specific, measurable, attainable, relevant, and time-limited (SMART) goals can increase the efficacity and adherence of the client (15). SMART defines the client's goals in a way that is precise and attainable in motivational interviewing (16). Also, to improve the quality of care provided, it is important to forward after-visit summaries and clarifying questions or discrepancies. Clients find it helpful to identify a clear schedule of follow-up visits to anticipate their next virtual visit and manage their co-created goals. These practices can benefit the client–provider relationship and therefore improve compliance with the treatment plan (14). The following is an example of a successful case study:

Patient A is a 50-y-old female, BMI 32 kg/m^2^, and has a history of hypertension with no other comorbidities. She was diagnosed with type 2 diabetes with a hemoglobin A1c (HbA1c) of 11.2%. During COVID-19, she has been extremely anxious and reaches out through Instagram for a virtual consultation to control her dietary intake and regulate her glucose levels. She receives a preconsultation questionnaire and guidelines for telenutrition consultations. She is billed through message, pays online, and then receives a video consultation through HIPAA-compliant tabibonline.com. Weekly video consultations are established for the first month with home body measurements and diabetes management education. After 5 mo of follow-up and a total of 8 virtual consultations, her HbA1c is 6% and she has attained a weight loss of 7 kg.

## Conclusion

With the current crisis, regulatory agencies in several countries are formally relaxing privacy and data protection regulations for video and other communications technologies. Most cite the “overwhelming public interest” in unprecedented times. Privacy laws have been changed and adapted to allow for telenutrition to the extent that it is covered by insurance companies (7, 8). Other agencies have started to develop simple remote tools to screen for diseases that in the past had to be screened through a face-to-face encounter (17).

In summary, virtual consultation in the nutrition practice is feasible and encouraged during the COVID-19 pandemic (8). In the long term, the e-nutrition clinic might become the norm for clinicians in building a successful therapeutic alliance with clients.

## Notes

The author reported no funding received for this study.

Author disclosure: The author reports no conflicts of interest.

Abbreviations used: COVID-19, coronavirus disease 2019; HbA1c, glycated hemoglobin; HIPAA, Health Insurance Portability and Accountability Act of 1996; SMART, specific, measurable, attainable, relevant, time-limited.
